# Autochthonous Cases of Mycetoma in Europe: Report of Two Cases and Review of Literature

**DOI:** 10.1371/journal.pone.0100590

**Published:** 2014-06-25

**Authors:** Dora Buonfrate, Federico Gobbi, Andrea Angheben, Stefania Marocco, Claudio Farina, Jef Van Den Ende, Zeno Bisoffi

**Affiliations:** 1 Center for Tropical Diseases (CTD), Sacro Cuore Hospital, Negrar, Verona, Italy; 2 Microbiology Institute, AO Papa Giovanni XXIII, Bergamo, Italy; 3 Department of Clinical Sciences, Institute of Tropical Medicine, Antwerp, Belgium; University of Brighton, United Kingdom

## Abstract

**Background:**

Mycetoma is a chronic granulomatous infection involving cutaneous and subcutaneous tissues. It is endemic in tropical and subtropical areas, but sporadic cases have been reported also in countries of temperate climate. The purpose of this paper is to review the cases of mycetoma in European subjects (and presumably acquired in Europe), to give an insight in the main factors associated with this condition, and to describe two previously unpublished cases observed at our Centre.

**Methods and Findings:**

PubMed was systematically searched for case reports and case series of mycetoma in Europeans reported between 1980 and 2014, using specific search strategies. Two further cases diagnosed by the authors are described. Forty-two cases were collected. Eleven cases were caused by *Scedosporium apiospermium*, mainly in immunosuppressed patients from Bulgaria, Germany, the Netherlands, Portugal, Slovenia, Spain and the United Kingdom. Excluding all patients with immunosuppression, 29 cases remain. Most of them were reported from Bulgaria and in Albanian patients (all diagnosed outside Albania). In the Bulgarian case series many different micro-organisms, both bacteria and fungi, were isolated, while all the 5 cases from Albania were caused by *Actinomadura* spp. Other countries reporting cases were Greece, Italy and Turkey. In general, *Actinomadura* spp is the most frequent causative agent isolated, followed by *Nocardia* spp and *Madurella mycetomatis*. The foot was the most reported site involved. Most patients were medically treated, but unfortunately a long-term follow up (at least one year) was available only in a few cases.

**Conclusions:**

Our review and our own cases suggest that Europeans without travel history can be affected by Madura foot. The lack of a surveillance system is likely to cause an underreporting of cases. Moreover, the unfamiliarity of Western doctors with this peculiar infection may cause a mismanagement, including unnecessary amputations.

## Introduction

### Rationale

Mycetoma is a chronic granulomatous infection involving cutaneous and subcutaneous tissues (with possible extension to fascia and bone), characterized by local swelling and sinuses draining pus and grains [1]. A variety of causative agents have been identified and, on the basis of the species isolated, the infection is classified as eumycetoma (in case of fungal agent) or actinomycetoma (in case of bacterial agent) [1]. Mycetoma causes high morbidity, and a proper management of the infection (including adequate identification of the causative agent) is essential in order to avoid a devastating progression of the disease and the need for amputation [2]. Although the global burden of mycetoma is not well defined, attempts to obtain a global estimation have been done [3]. Areas of endemicity are present in tropical and subtropical regions, but occasionally cases from countries with a temperate climate have been reported [3].

### Objectives

Aim of this review is to identify the cases of mycetoma reported in European patients who presumably acquired the infection in Europe, and to give an insight in factors associated with this condition. Moreover, two further cases of mycetoma diagnosed in two Albanian patients attending our Centre are described.

### Case report 1

Patient 1 was a 43 year-old man born in Albania, where he had worked as forest ranger. Arrived in Italy with one of the first waves of migration of undocumented Albanian people in 1992, he presented in 1997 to a provincial hospital of Veneto Region with a grossly swollen right foot with sinuses on the right medial malleolus, draining white granules. Referred to the Orthopedic Department, he was scheduled for amputation, with no specific diagnosis. The day of hospital admission, the surgeon in charge, who had not previously seen the patient, decided to refer him to the Centre for Tropical Diseases (CTD) of Negrar (Verona), for advice prior to surgery. The patient reported increasing swelling and secretion for about 12 years. During this period he had been treated both with oral (unspecified) antibiotics and with attempts to surgical debridement in his country and then in Italy, with no specific diagnosis. Despite these treatments, in recent months the number of sinuses had markedly increased and so had the drainage of white grains, hence the decision to amputate. At CTD, *Actinomadura madurae* was isolated from the granules, so a specific antibiotic treatment was prescribed, according to recommendations by Welsh et al [4]. The patient was treated for about 24 months, obtaining complete cure and no recurrence during the 16 year-follow up (last visit in October, 2013).

### Case report 2

Patient 2, a 46 year-old Albanian woodcutter, was a colleague and friend of patient 1 but had not dared to travel to Italy with him. He had started presenting signs of mycetoma on the right foot in 1982. After receiving several courses of antibiotics (penicillin plus gentamycin followed by other unspecified drugs) without response, he underwent a below-knee amputation in 1995. Nevertheless, the disease showed further progression, so an above-knee amputation was proposed, but he refused. He presented to the CTD in 2001, the year of his arrival in Italy. He presented a swollen stump with nodules and sinuses draining white granules. The specimens obtained through needle aspiration permitted to diagnose, again, a mycetoma due to *Actinomadura madurae*. The patient was successfully treated with antibiotic therapy, similarly to patient 1, and showed no recurrence during the 2 year-follow up (last visit in November, 2003, then he came back to Albania).

## Methods

### Eligibility criteria

A literature survey and analysis was conducted to collect the cases of mycetoma reported in Europe between 1980 and 2014.

### Information sources and search

PubMed database was searched for case reports and case series, limiting the search to humans and to the following languages: English, Spanish, French, Italian and Portuguese. Search terms used were: Mycetoma OR Madura's foot AND Europe OR Austria OR Belgium OR Bulgaria OR Croatia OR Cyprus OR Czech OR Denmark OR Estonia OR Finland OR France OR Germany OR Greece OR Hungary OR Ireland OR Italy OR Latvia OR Lithuania OR Luxemburg OR Malta OR Netherlands OR Poland OR Portugal OR Romania OR Slovakia OR Slovenia OR Spain OR Sweden OR United Kingdom OR Iceland OR Montenegro OR Serbia OR Macedonia OR Turkey OR Albania OR Bosnia OR Kosovo. The list of countries included in the search strategy is based on the lists of countries present on the European Union website (“member countries” + “on the road to EU membership” + “potential candidates”). PubMed search was done on the 20th December 2013.

### Study selection and data collection process

Inclusion criteria: diagnosis performed in Europe in European citizens. We developed a data extraction sheet, pilot-tested it on five randomly-selected included papers, and refined it accordingly. One review author extracted the data from included studies and the second author checked the extracted data. Disagreements were resolved by discussion between the two review authors; if no agreement could be reached, it was planned a third author would decide.

### Data items

Information was extracted from each included paper on: (1) characteristics of patients (including country of origin, underlying conditions, concomitant therapies); characteristics of infection (including infectious agent, site of infection); (3) type of intervention (including medical or surgical interventions); (4) type of outcome measure (including complete healing, improvement, relapses, length of follow up, unintended effects of treatment); (5) country where the diagnosis was done and reported.

## Results

### Study selection

Our search strategy permitted to identify 157 papers, of which 111 were excluded by title and abstract evaluation. Full-text papers were then assessed for eligibility according to the given criteria. Eventually, 23 papers were included, reporting 39 mycetoma cases. Moreover, one paper from grey literature and the two cases described in this paper were added, resulting in a total of 42 cases (See PRISMA flow chart –Figure S1).

### Study characteristics

**Figure 1:**
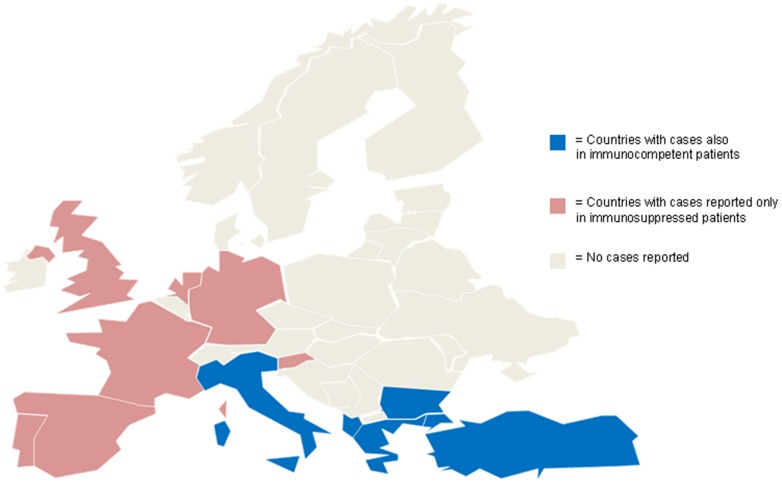
Map showing the countries of origin of all the patients with mycetoma.

Countries of origin of patients are represented in Figure 1. Twelve of the cases were infections in patients with immunosuppression (either caused by chronic or transient iatrogenic conditions). The causative organism was *Scedosporium apiospermium* in 10/12 cases [5]–[14]. In the other two patients the species isolated were *Exophiala jeanselmei* [15] and *Arthrographis kalrae* [16] (the latter was defined as the “first case” of mycetoma caused by this agent). *Scedosporium apiospermium* was isolated also in a patient included in a case series from Bulgaria [17], but information on the immune status of the patient is lacking. In a case series from the UK, a case of mycetoma in a patient born in that country was reported, clinical and microbiological findings were not reported [18].

**Figure 2:**
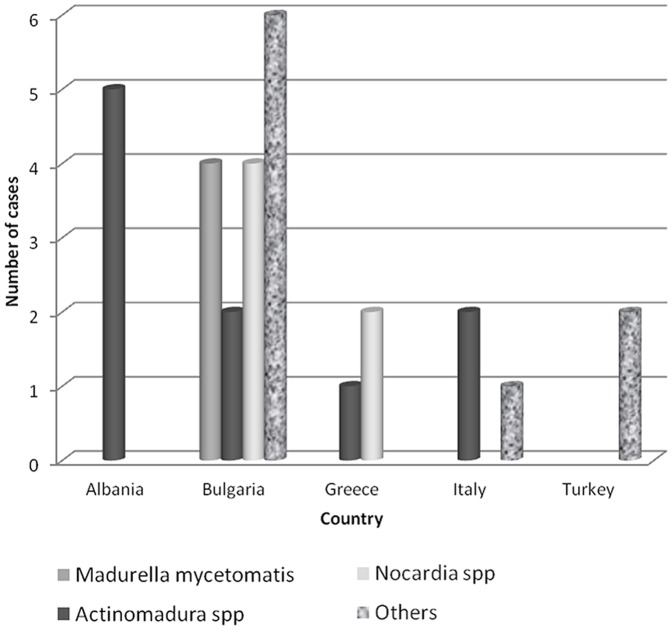
Number of cases in which each species was isolated per country.

Excluding this latter case because of lack of information and the above referred reports on immunosuppressed patients, the number of countries reporting cases is reduced to 5, all in South - Eastern Europe (countries represented in blue in Figure 1): Albania, Bulgaria, Greece, Italy, and Turkey. Figure 2 resumes the number of cases by species per country.

The cases in Albanian patients (including the two cases described in this paper) were diagnosed and reported in Greece [19], [20] and in Italy [21]. All 5 patients had an infection caused by *Actinomadura madurae.* Of these 5 patients, 4 were treated exclusively with antibiotic therapy, only our patient 2 also had surgery. De Palma et al. describe an improvement 6 months after treatment [21]. As for the cases described in Greece, one patient was lost to follow up about 5 weeks after the beginning of the treatment [19], while there are no details about the follow up of the other patient [20].

All Bulgarian cases are reported in a case series by Balabanoff [17] published in 1980. A variety of causative agents is described, both fungal and bacterial. One patient with *Madurella mycetomatis* infection had the infected foot amputated. There is no information about the post-treatment follow up of the patients.

There were 3 patients of Greek origin. One patient was injured during a motorcycle accident in Crete, and developed an infection due to *Nocardia asteroides* (although also *Sporothrix schenckii* was isolated from the material obtained from the wound) [22]. Another Greek patient had a mycetoma on the forehead caused by *Nocardia brasiliensis* [23] and the third one developed a mycetoma of the foot caused by *Actinomadura madurae* [24]. All these patients received specific medical treatment and no surgery was required. The authors report improvement of the infections from 7 to 9 months after treatment (no longer follow up is available).

Two case reports describe mycetoma of the foot caused by *Actinomadura madurae* in Italian women [25], [26]. Both patients had relapses/progression of the disease despite antibiotic therapy based on the antibiograms. After a further antibiotic course, one patient resulted stationary [26] and the other one improved [25] (but both cases were described shortly after the last therapeutic course). The third case was caused by *Fusarium moniliforme* and affected the foot of a retired miner [27].

Cases in Turkish patients were reported from Turkey [28] and from the UK [18]. In the first one, no causative agent was isolated from the material draining from the foot, while in the second one a mycetoma of the hand sustained by *Streptomyces somaliensis* was described. The latter case was treated with surgical intervention plus medical therapy for 10 months, obtaining resolution after one year.

### Results of individual studies

Table 1 summarizes the number of cases reported for the most frequent agents isolated, with the body site involved, therapy administered and outcome.

**Table 1:** 

Causative agent	N. of cases	Site	Therapy	Outcome*
*Actinomadura* spp	10	All: foot	8 cases: medical; 1 amputation	1 complete response**; 2 relapses
*M. mycetomatis*	4	3 foot; 1 hand	Missing data	1 amputation
*Nocardia* spp	5	4 foot; 1 forehead	2 medical	Missing data

*If available, at least 1-year follow up.

**The patient with the amputated limb was then treated with antibiotics with complete response.

## Discussion

### Summary of evidence

In our review part of the cases reported in Europeans were associated to immunosuppression (mostly caused by *Scedosporium apiospermium*), which is unusual in “classical” mycetoma, although it has been implicated in the dissemination from the primary site [2]. Several “classical” cases also were diagnosed, all in patients originating from the Southern-East part of the continent. This geographical distribution might be due to climatic factors favorable to the survival of the most common causative agents found (*Actinomadura* spp, *Madurella mycetomatis* and *Nocardia* spp) [2], [3]. Also socioeconomic issues might be implicated, considering that almost all the reported infections affected the foot (barefoot walking?) and that the number of reports decreases through the years (improvement of quality of life?). In general, a conservative management, based on microbiology and specific medical treatment was the preferred approach, which was generally appropriate as most cases were actinomycetomas [1], [2]. Only one of the Bulgarian and one of the Albanian patients had their foot or leg amputated. In the latter case (our patient 2) amputation could have been avoided, and the medical therapy for *Actinomadura madurae* administered afterwards allowed a complete cure.

### Limitations

The small number of case reports found in literature does not necessarily reflect a decreased incidence of mycetoma, because the lack of a surveillance system and the unfamiliarity of Western physicians with this condition may have caused a substantial under reporting. For instance, cases from Bulgaria are limited to a case series published in 1980, but it cannot be excluded that more cases occurred since. Moreover, cases identified in Albanian patients were all reported in Greece or Italy; presumably more cases must have occurred in Albania, but they were either not diagnosed or not published.

While the clinical characteristic of the lesions and microbiology results were generally described, we found less details about therapy and especially about outcome. Many reports were published just few months after diagnosis, thus the follow up was not long enough to prove a full recovery. For this purpose we considered only cases with at least 1 year of follow up, although this might not be enough if we consider possible recurrences and cases requiring treatment for months or years [1].

## Conclusions

Though rare, mycetoma can occur in temperate countries of Europe, where most physicians are unfamiliar with this condition, with risk of under reporting and, much more importantly, of mismanagement.

## Supporting Information

Figure S1 **PRISMA Flow Chart.**
(TIF)

Table S1 **PRISMA Checklist.**
(DOC)
