# Supplementary figures and images for: Autochthonous Cases of Mycetoma in Europe: Report of Two Cases and Review of Literature
Source: PLoS One. 2014 Jun 25;9(6):e100590. doi: 10.1371/journal.pone.0100590 (PMC4070928; doi:10.1371/journal.pone.0100590)

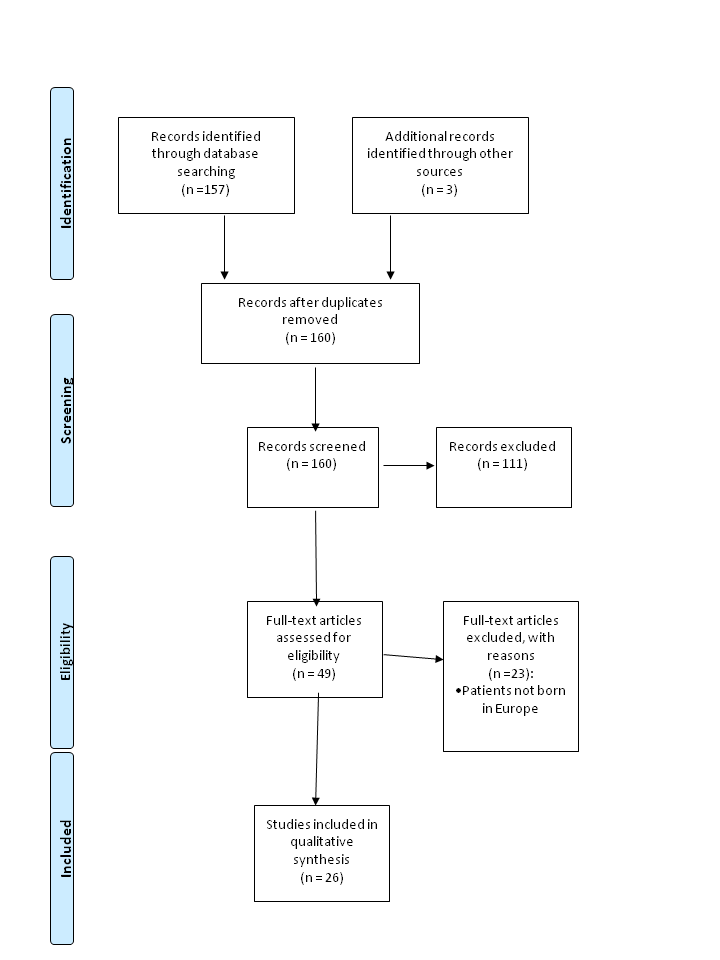

Supplement: Figure S1 — PRISMA Flow Chart. (TIF) [file pone.0100590.s001.tif]
